# Promotion of Th1 and Th2 responses over Th17 in *Riemerella anatipestifer* stimulation in chicken splenocytes: Correlation of gga-miR-456-3p and gga-miR-16-5p with NOS2 and CCL5 expression

**DOI:** 10.1371/journal.pone.0294031

**Published:** 2023-11-06

**Authors:** Paula Leona T. Cammayo-Fletcher, Rochelle A. Flores, Binh T. Nguyen, Andrea Gail M. Villavicencio, Seung Yun Lee, Woo H. Kim, Wongi Min

**Affiliations:** College of Veterinary Medicine & Institute of Animal Medicine, Gyeongsang National University, Jinju, Korea; University of Michigan Medical School, UNITED STATES

## Abstract

*Riemerella (R*.*) anatipestifer* poses a significant threat to ducks, resulting in mortality rates ranging from 5–75%. This disease is highly infectious and economically consequential for domestic ducks. Although other avian species, such as chickens, also display susceptibility, the impact is comparatively less severe than in ducks. IL-17A has a pronounced correlation with *R*. *anatipestifer* infection in ducks, which is less in chickens. This study performed an *in vitro* transcriptome analysis using chicken splenic lymphocytes collected at 4-, 8-, and 24-hour intervals following *R*. *anatipestifer* stimulation. The primary objective was to discern the differentially expressed genes, with a specific focus on IL-17A and IL-17F expression. Moreover, an association between specific miRNAs with NOS2 and CCL5 was identified. The manifestation of riemerellosis in chickens was linked to heightened expression of Th1- and Th2-associated cells, while Th17 cells exhibited minimal involvement. This study elucidated the mechanism behind the absence of a Th17 immune response, shedding light on its role throughout disease progression. Additionally, through small RNA sequencing, we identified a connection between miRNAs, specifically miR-456-3p and miR-16-5p, and their respective target genes NOS2 and CCL5. These miRNAs are potential regulators of the inflammatory process during riemerellosis in chickens.

## Introduction

*Riemerella (R*.*) anatipestifer*, a member of the *Flavobacteriaceae* family, causes the condition known as riemerellosis, characterized by acute to chronic septicemia. While its primary hosts are domestic ducks and geese, susceptibility to *R*. *anatipestifer* infection extends to other avian species, such as chickens [1–3].

Despite the economic significance of this disease and the poultry being the second source of meat consumption next to pork, there are relatively few studies regarding host immune responses against *R*. *anatipestifer* infection. Zhou et. al. conducted a study that identified immune and inflammatory response genes in ducks [4]. Similarly, Fernandez et. al. reported upregulated expression of IL-17 and IL-6, which are associated with Th17-cell differentiation in *Riemerella-*infected duck splenic lymphocytes, although to a lesser extent in chicken splenic lymphocytes [5]. Furthermore, chickens exhibited higher expression of Th1 and Th2 cells following *R*. *anatipestifer* infection than did ducks. Additionally, the upregulation of IL-1β and IL-6, along with IL-17A, strongly indicates an association of IL-17A in immune responses against *R*. *anatipestifer* both *in vivo* and *in vitro* in ducks, and to a lesser extent in chickens. This finding gains further support from the observed downregulation of inflammatory cytokines in *R*. *anatipestifer*–infected ducks treated with berberine [6] and 3,3’-diindolylmethane [7].

Inflammation is a pathophysiological consequence of riemerellosis, resulting in characteristic pathognomonic signs in avian species, including fibrinous meningitis, pleural effusion, and exudative infiltrates in air sacs and the liver [2]. In many inflammatory disorders, NOS2 and CCL5 are upregulated, promoting the synthesis of IL-6 and IL-8. NOS2 is upregulated in *R*. *anatipestifer–*infected ducks [8] and during bacterial infections such as *Salmonella Typhimurium* in chickens [9]. Another immune-related gene associated with chicken inflammatory disorders is CCL5, which is upregulated during infections of *Clostridium perfringens* and *Eimeria* [10].

Traditional small RNAs are non-coding RNA molecules with a nucleotide of less than 200 in length and includes PIWI interacting RNA (piRNA), small interfering RNA (siRNA) and microRNA (miRNA) [11]. miRNAs, as post-transcriptional gene regulators, represent a class of non-coding RNAs that are pivotal in diverse biological and pathological processes, with a profound impact on host immune responses [12,13]. In stark contrast to traditional small drug molecules that target a limited spectrum of proteins or merely interact with cell-surface receptors, miRNAs exert their influence through mRNA degradation, leading to gene downregulation [12]. While miRNAs have been extensively studied as prognostic biomarkers or therapeutic targets in human contexts [13,14], their roles in avian diseases remain largely unexplored. The miRBase database, version 22.1 [15], documents approximately 1232 mature miRNAs within the chicken genome.

Despite the scarcity of functional studies on chicken miRNAs, existing reports highlight the regulatory potential of gga-miR-456-3p (miR-456-3p) [16–18] and gga-miR-16-5p (miR-16-5p) [19,20] in immune-related genes implicated in poultry physiological and disease processes. However, their roles during *R*. *anatipestifer* infection have yet to be explored.

In this study, we employed high-throughput RNA-seq to conduct a comprehensive transcriptome analysis of chicken splenic lymphocytes following *R*. *anatipestifer* treatment at different time points. Our primary focus was the identification of differentially expressed genes (DEGs), with specific attention directed toward IL-17A and IL-17F expression. Furthermore, this investigation delved into the functional and biological roles of miR-456-3p and miR-16-5p as potential regulators of the inflammatory process during *R*. *anatipestifer* infection in chickens. These miRNAs were studied in the context of their interactions with their respective target genes NOS2 and CCL5, alongside other inflammatory cytokines influenced by these genes. Analysis of this dataset could facilitate a comparative examination of gene expression within chicken splenic lymphocytes, both in the presence and absence of *R*. *anatipestifer* stimulation. Furthermore, this analysis aimed to validate existing observations while offering novel perspectives into the intricate molecular mechanisms underpinning *R*. *anatipestifer* infection and its interaction with the host immune system.

## Materials and methods

### Animals and ethics statement

ROSS308 broiler chicks (Samhwa, Hongseong-gun, Korea) were reared in wire cages under a temperature-controlled environment with unlimited access to anticoccidial/antibiotic-free feed and water. Chickens were sacrificed using atlanto-occipital dislocation. All animal maintenance and experimental procedures followed the Gyeongsang National University Guidelines for the Care and Use of Experimental Animals and were approved by the Institutional Animal Care and Use Committee (GNU-221011-E0132).

### Cell culture and infection

Chicken splenic lymphocytes were isolated as previously described [21]. The harvested cells were cultured in DMEM (Gibco Life Technologies, NY, USA) supplemented with 1% penicillin-streptomycin (10,000 unit/ml) and 10% fetal bovine serum under 5% CO_2_ at 41°C. Suspensions of splenic lymphocytes were prepared at a concentration of 5 × 10^6^ cells/ml, and subsequently stimulated with heat-killed *R*. *anatipestifer* serotype 1 (1 × 10^6^ CFU/ml) for 4, 8, or 24 hours as previously described [5,6]. The heat-killed *R*. *anatipestifer* was prepared through a 5-min incubation in a heating block set to 100°C.

### *In silico* mRNA-seq and data analysis

Construction of small RNA (smRNA) libraries was carried out using a TruSeq DNA PCR-Free LT Library Prep Kit, followed by sequencing on an Illumina NovaSeq 6000 platform (Egenome, Seoul, Korea). For the acquisition of high-quality reads, the FastQC v0.11.8 program was applied to filter Fast-Q formatted raw reads. Trimmomatic v0.39 was utilized to trim off the adapter sequences and sequences with low-quality scores below 20 from the raw data.

The resulting pool of high-quality raw reads was mapped to the *Gallus gallus* (GRCg6a) reference genome utilizing HISTAT2. Subsequently, FeatureCount was employed to quantify the mapped reads concerning their respective genomic features. Differential expression analysis was conducted using edgeR.

### Next-generation sequencing and miRNA data analysis

The construction and sequencing of smRNA libraries were performed utilizing SMARTer smRNA-Seq Kits on the Illumina platform (Macrogen, Seoul, Korea). To ensure data quality, raw read files in FAST-Q format underwent filtration based on a minimum of 20% bases with a phred quality score of ≥20 using FastQC v0.11.7.

Trimming of raw sequence reads to remove adapter sequences was carried out using Cutadapt 2.8 [22]. Comprehensive miRNA profiling was achieved through short- and long-target analyses. Specifically, trimmed reads and non-adapter reads within the range of ≥ 18 bp to ≤ 50 bp were removed for long-target analysis (≥ 50), while trimmed reads were used for short target analysis (< 50 bp).

High-quality clustered reads were then aligned to the *Gallus gallus* (GRCg6a) reference genome using miRBase v22.1 [23] to facilitate the identification of both mature miRNAs and pre-miRNAs. Furthermore, alignment to the non-coding RNA database was conducted using RNAcentral v14.0, leveraging Bowtie 1.1.2 software [24] to effectively identify and exclude small nuclear RNA, ribosomal RNA, transfer RNA, and small nucleolar RNA.

### Analysis of differentially expressed miRNAs (DE-miRNAs)

DE-miRNAs between the infected and control groups were examined across different time points using various statistical approaches, including fold change and exactTest within the edgeR framework for each paired comparison at every time point. DE-miRNAs were identified using t-tests with fold changes ≥ 2 and *p-*values < 0.05 were considered statistically significant.

### Functional analysis of target genes

To elucidate potential immune-related target genes regulated by DE-miRNAs, the miRDB database [25] was employed. Matched genes obtained from the RNA-seq results were subjected to further analysis. For comprehensive functional analysis, Gene Ontology (GO) and Kyoto Encyclopedia of Genes and Genomes (KEGG) Pathway analyses were conducted using Panther 16.0 [26]. This analysis specifically centered around Th17-related cytokines or pathways, namely NOS2 and CCL5. To pinpoint precise binding sites for miR-456-3p and miR-16-5p on NOS2 and CCL5, respectively, we employed the RNA hybrid computation tool [27]. This enabled accurate prediction of binding interactions between these miRNAs and their target genes.

### Validation of gene expression

RNA and miRNA selections were guided by specific parameters. Initially, immune-related genes were chosen from the list of DE-mRNAs identified in the RNA sequencing data. These selected genes were then cross-referenced with their corresponding predicted target miRNAs from miRDB. Subsequently, the target miRNAs were matched with the significant DE-miRNA list obtained from the miRNA-seq data. For validation purposes, two miRNAs were selected based on their association with two specific genes. These genes, in turn, were linked to other genes, forming an interconnected network. All selected genes underwent quantitative reverse transcription PCR (qRT-PCR) analysis.

Total RNA and miRNA extraction was performed using RiboEx Total RNA Isolation Solution (GeneAll, Seoul, Korea). Purification was carried out using either an RNeasy Mini Kit (Qiagen, Hilden, Germany) for RNA or a miRNeasy Tissue/Cells Advanced Micro Kit (Qiagen, Hilden, Germany) for miRNA. Subsequently, cDNA synthesis utilized a QuantiTect Reverse Transcription Kit (Qiagen, Hilden, Germany) with a random hexamer primer for mRNA and a miRCURY LNA RT Kit (Qiagen, Hilden, Germany) for miRNA.

qRT-PCR was conducted using an AccuPower 2X Green Star qPCR Master Mix (Bioneer, Daejon, Korea) for mRNA and a miRCURY LNA SYBR Green PCR Kit (Qiagen, Hilden, Germany) for miRNA. Reactions were performed on a CFX96 RealTime PCR System (Bio-Rad, CA, USA) with specific primers. Relative expression levels were quantified using the comparative ΔΔCT method, using either β-actin or U6 as an internal control for normalization. The log_2_ fold change in relative expression from *R*. *anatipestifer*–stimulated samples was calculated relative to the expression levels of unstimulated splenic lymphocytes at the same time points. This approach facilitated a straightforward comparison with the miRNA-seq and RNA-seq results. Using the miRprimer2 software [28], primer sequences were generated for the selected miRNAs, namely gga-miR-456-3p and gga-miR-16-5p. The primer sequences used are listed in Table 1.

**Table 1:** Primer sequences for qRT-PCR analysis confirmation in *R*. *anatipestifer*–infected chicken splenic lymphocytes.

Gene		Sequence (5’-3’)	References
U6	F	GGAACGATACAGAGAAGATTAGC	[29]
	R	TGGAACGCTTCACGAATTTGCG	
gga-miR-456-3p	F	GCAGGCTGGTTAGATGGT	(This study)
	R	GGTCCAGTTTTTTTTTTTTTTTGACA	
gga-miR-16-5p	F	CAGTAGCAGCACGTAAAT	(This study)
	R	GGTCCAGTTTTTTTTTTTTTTTCAC	
β-actin	F	CACAGATCATGTTTGAGACCTT	[30]
	R	CATCACAATACCAGTGGTACG	
NOS2	F	TGGGTGGAAGCCGAAATA	[31]
	R	GTACCAGCCGTTGAAAGGAC	
CCL5	F	CAGCAAATGCCCACAGG	[32]
	R	TGCAGCTCCAGGAAGTTGAT	
IL-6	F	CAAGGTGACGGAGGAGGAC	[30]
	R	TGGCGAGGAGGGATTTCT	
IL-10	F	CGGGAGCTGAGGGTGAA	[30,33,34]
	R	GTGAAGAAGCGGTGACAGC	
IL-1β	F	TGGGCATCAAGGGCTACA	[30,33,34]
	R	TCGGGTTGGTTGGTGATG	
IL-17C	F	AGCCTCACGAGAGATCCATC	[35]
	R	CCTCCCTGTCTTCACATCCAC	
CXCLi1	F	CCTCACTGCAAGAATGTGGAAG	[36]
	R	TATTGGTGTCTGACTTGTCCAG	
CXCLi2	F	TGGTTTCAGCTGCTCTGTCG	[36]
	R	AAGCACACCTCTCTTCCATCC	
IL-13RA2	F	CACCATCTCCAGAGCAAATCG	[37]
	R	AAGCCCTCATCAGCACAGAAG	
IL4I1	F	GGAGAAGGACTGGTATGTGGAG	[38]
	R	GCTTCAGGTCAAACTGCCTTAT	
IL-1R2	F	GTTTGGTTGGGTTGTGGTTGG	[39]
	R	TGACAACTGGATTAGGCTCTGG	
IL-17A	F	GAGAAGAGTGGTGGGAAAG	[40]
	R	TCTACAAACTTGTTTATCAGCAT	
IL-17F	F	TGAAGACTGCCTGAACCA	[30]
	R	AGAGACCGATTCCTGATGT	

### Functional validation of miRNA mimics and cell transfection

Preliminary experiments showed that the macrophage-derived HD11 cell line was more sensitive than CU91 (T-cell line) stimulated with *R*. *anatipestifer*. Thus, the HD11 cell line was cultured in 6-well plates containing DMEM (Gibco Life Technologies, NY, USA) supplemented with 1% penicillin-streptomycin (10,000 unit/ml) and 10% fetal bovine serum. Cells were incubated under 5% CO_2_ at 41°C. Cell suspensions were prepared at a concentration of 5 × 10^6^ cells/ml and allowed to attach overnight.

Stimulation was initiated by exposing cells to heat-killed *R*. *anatipestifer* serotype 1 (1 × 10^6^ CFU/ml) for 4 hours. Concurrently, transfection was carried out using a miR-456-3p mimic with the sequence ACAACCAUCUAACCAGCCUGTT and a miR-16-5p mimic with the sequence CCAAUAUUUACGUGCUGCUATT. Both negative control (miR-NC mimic) which has a distinct sequence fairly different from targeted miRNAs and positive (miR-PC mimic) which has an almost identical sequence to the targeted miRNAs were also included. All miRNA mimics were manufactured by Ambion® Life Technologies, Seoul, Korea. The transfection process utilized Lipofectamine® RNAiMAX (Invitrogen, MA, USA) as the transfection reagent.

### Statistical analysis

All *in vitro* experiments were conducted in triplicate. Group comparisons were assessed through either Student’s t-test or one-way ANOVA with post hoc analysis of Dunnet’s test, employing InStat statistical software (GraphPad, CA, USA). Data are presented as mean ± standard error of the mean (SEM). Statistical significance was defined as a *p*-value <0.05. For visualization purposes, log_2_ fold change values were utilized.

## Results

### *In silico* analysis and identification of DE-mRNAs during *R*. *anatipestifer* stimulation

**Fig 1:**
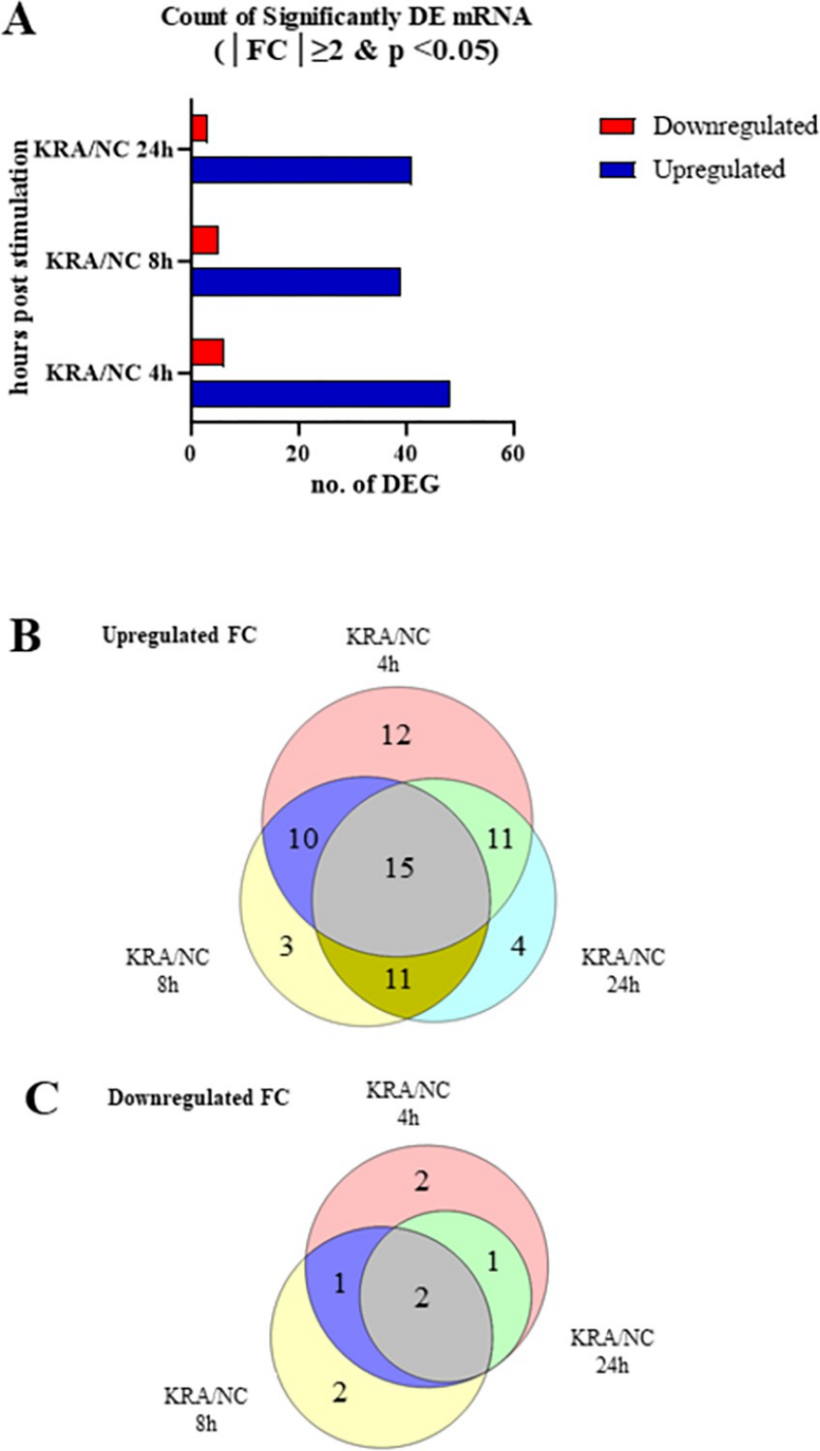
Comparative analysis of differentially expressed mRNAs (DE-mRNAs). Six pooled sets of chicken splenic lymphocyte samples were collected at 4-, 8-, and 24-hour time points from both non-infected control (NC) and heat-killed *R*. *anatipestifer* (KRA)-stimulated samples. Differentially expressed gene identification and normalization revealed fold changes (FC) in both upregulated and downregulated mRNA pairs. The bar graph (A) illustrates significant fold changes for DE-mRNAs between KRA and NC samples across the time points. A Venn diagram was constructed to illustrate the relationships and numbers of (B) upregulated and (C) downregulated DE-mRNAs across time points.

Among the initial 18,492 genes identified through mRNA-seq, only 157 mRNAs exhibited significant differential expression with an FDR of *p* <0.05. Individually computed logFC wherein |FC| ≥2 was used to represent for the total DEGs on the venn diagram on Fig 1. The 4-hour time point displayed the highest number of both upregulated and downregulated mRNAs, followed by 24 hours, and then 8 hours with the fewest upregulated and downregulated mRNAs. Common to all three time points, 15 mRNAs were consistently upregulated, while 2 mRNAs were commonly downregulated. The relationship between the significant DE-mRNAs and their temporal occurrence is depicted in Fig 1.

From the RNA-seq analysis, a list of differentially expressed genes common to three time points (Table 2) and a list of common immune-related genes were generated (S1 Table). A subset of this list, including NOS2, CCL5, and other immune-related genes, was created (Table 3). NOS2 showed significant upregulation across all time points, whereas CCL5 displayed significant upregulation at 4 hours and moderate upregulation without significance at the other time points. The remaining immune-related genes associated with NOS2 and CCL5 demonstrated consistent upregulation throughout all time points. Significant differences are expressed in arrows ↑ for upregulated and ↓ for downregulated genes (|FC| ≥2, *p* <0.05).

**Table 2:** List of differentially expressed genes common to three time points from RNA-seq results.

Gene	Description	Function	Log_2_FC (4h)	Log_2_FC (8h)	Log_2_FC (24h)
*LOC422654*	Chemokine (C-X-C motif) ligand 1-like	Chemoattractant of neutrophils or other non-hematopoietic cells to the site of infection	↑3.97	↑ 3.87	↑ 4.27
*NOS2*	Nitric oxide synthase-2	Involved in inflammation by enhancing synthesis of proinflammatory mediators like IL6 and IL8	↑ 3.41	↑ 3.18	↑ 3.09
*TNIP3*	TNFAIP3-Interacting Protein 3	Inhibits NF-kappa-B activation induced by TNF, TLR4 and IL-1. Also involved in IL-23 signaling pathway	↑ 2.42	↑ 2.41	↑ 2.85
*SLCO4C1*	Solute Carrier Organic Anion Transporter Family Member 4C1	Mediates the transport of organic anions such as steroids and numerous drugs.	↑ 2.42	↑ 2.66	↑ 3.09
*IL13RA2*	Interleukin-13 receptor subunit alpha-2	IL-13 receptor involved in regulation of allergic asthma and eosinophilic inflammation	↑ 2.1	↑ 3.12	↑ 2.73
*MMP10*	Matrix Metallopeptidase 10	Degrades extracellular matrix in normal physiological processes	↑ 3.43	↑ 2.52	↑ 2.2
*STEAP4*	STEAP4 Metalloreductase	Functions as a metalloreductase	↑ 4.00	↑ 3.43	↑ 2.92
*STEAP1*	STEAP Family Member 1	Functions as a metalloreductase	↑ 3.09	↑ 3.53	↑ 3.3
*MMP27*	Matrix Metallopeptidase 27	Degrades extracellular matrix in normal physiological processes	↑ 2.47	↑ 4.89	↑ 2.84
*LOC419276*	Bactericidal permeability-increasing protein-like	Antibacterial response	↑ 4.70	↑ 2.64	↑ 2.03
*CYP1A1*	Cytochrome P450 Family 1 Subfamily A Member 1	Catalyze many reactions involved in drug metabolism and synthesis of cholesterol, steroids and other lipids.	↑ 4.30	↑ 3.08	↑ 2.78
*IL1B*	Interleukin-1 beta	Proinflammatory cytokine, neutrophil chemotaxis, T-cell activation and production, B-cell activation and antibody production, promotes Th17-cell differentiation	↑ 3.70	↑ 2.06	↑ 2.15
*CXCL14*	C-X-C Motif Chemokine Ligand 14	Homeostasis of monocyte-derived macrophages	↑ 2.24	↑ 2.68	↑ 2.3
*LOC121107512*			↑ 4.70	↑ 2.64	↑ 2.03
*SLC7A13*	Solute Carrier Family 7 Member 13	Mediates exchange transport for L-cystine.	↓ -2.35	↓ -2.04	↓ -2.08
*LOC121113084*	Oxygen-regulated protein 1-like		↓ -2.14	↓ -3.00	↓ -2.29

FC: Fold change; (↑): Upregulated. (↓): Downregulated, |FC| ≥2, *p* <0.05.

**Table 3:** Differentially expressed NOS2, CCL5 and other immune-related genes from RNA-seq results.

Gene	Description	Function	Log_2_ FC (4 h)	Log_2_ FC (8 h)	Log_2_ FC (24 h)
*NOS2*	Nitric oxide synthase 2	Involved in inflammation, enhances synthesis of proinflammatory mediators like IL6 and IL8	↑ 3.41	↑ 3.18	↑ 3.09
*CCL5*	C-C motif chemokine ligand 5	Chemoattractant for monocytes, memory T cells, and eosinophils	↑ 2.02	1.74	0.38
*IL6*	Interleukin 6	Proinflammatory cytokine, induces B-cell maturation	↑ 2.67	1.84	↑ 2.15
*IL1B*	Interleukin-1 beta	Proinflammatory cytokine, neutrophil chemotaxis, T-cell activation and production, B-cell activation and antibody production, promotes Th17-cell differentiation	↑ 3.7	↑ 2.06	↑ 2.15
*IL1R2*	Interleukin 1 receptor type 2	Non-signaling receptor for IL-1A, IL-1B, and IL1RN, reduces IL-1B activity through competitive binding	↑ 2.47	1.55	1.18
*IL4I1*	Interleukin 4 induced 1	Inhibits T-cell activation/proliferation, promotes CD4+ T-cell differentiation to FOXP3+ Tregs, regulates B cells	1.69	↑ 2.38	↑ 2.06
CXCLi1	Interleukin 8-like 1	Chemotactic for neutrophils to site of infection	↑ 3.73	1.85	↑ 3.1
CXCLi*2*	Interleukin 8-like 2	Chemotactic for neutrophils to site of infection	↑ 2.94	1.56	↑ 2.64
*IL10*	Interleukin 10	Immune regulatory cytokine	↑ 4.55	1.39	↑ 2.58
*IL13RA2*	Interleukin-13 receptor subunit alpha-2	IL-13 receptor involved in regulation of allergic asthma and eosinophilic inflammation	↑ 2.1	↑ 3.12	↑ 2.73
*IL17C*	Interleukin 17C	Promotes release of TNFa and IL-1B from a monocytic cell line	1.63	↑ 2.66	↑ 2.84

FC: Fold change; (↑): Upregulated. (↓): Downregulated, |FC| ≥2, *p* <0.05.

### Temporal modulation of Th17 signaling shapes IL-17A expression dynamics during *R*. *anatipestifer* stimulated splenocytes

**Fig 2:**
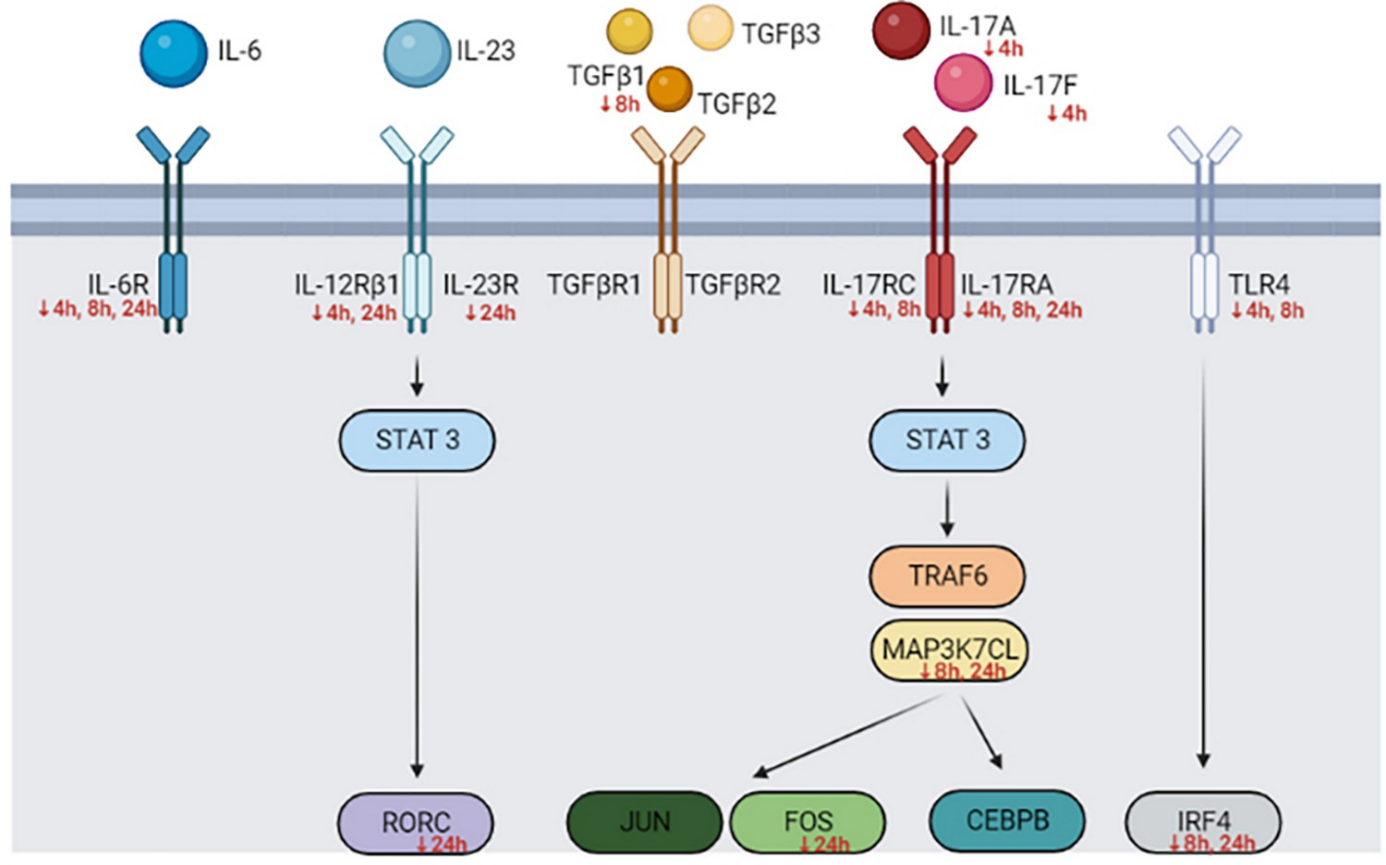
Th17 signaling pathway during *R*. *anatipestifer* infection in chickens. Utilizing the differentially expressed gene list from the RNA-seq results, a set of IL-17A-related genes was extracted to elucidate the downstream pathway of Th17 signaling. IL-6, IL-23, TGF-β1, TGF-β2, and TGF-β3 induce STAT3 phosphorylation through their respective receptors. Subsequently, STAT3 regulates RORC expression. Activation of STAT3 is also induced by binding of IL-17A and IL-17F to their receptors. TRAF6 and MAP3K7CL mediate the activation of AP-1 (JUN and FOS) and CEBPB. Additionally, TLR4 binding triggers IRF4 activation. Red arrows (↓) indicate downregulation at specific time points, while other genes are consistently upregulated across all time points.

A schematic diagram (Fig 2) was constructed to depict the downstream events of the Th17 precursors pathway, focusing on the genes associated with IL-17A. Across all time points, both the precursor IL-6 receptor (IL-6R) and the IL-17A receptor (IL-17RA) were downregulated. Notably, certain genes demonstrated reduced expression at two distinct time points, including IL-12RB1 at 4 and 24 hours, IR4 and MAP3K7CL at 8 and 24 hours, and IL-17RC at 4 and 8 hours. In contrast, several genes were exclusively downregulated at specific time points, such as IL-17A and IL-17F at 4 hours, TGFB1 at 8 hours, and IL-23R, RORC, and FOS at 24 hours. Detailed gene expression values are presented in Table 4. These observations underscore the pivotal factors contributing to the limited expression of the Th17 response during *R*. *anatipestifer* infection in chickens.

**Table 4:** Expression of IL-17A-related genes at different time points.

Gene	Description	Function	Log_2_ FC (4 h)	Log_2_ FC (8 h)	Log_2_ FC (24 h)
*IL17A*	Interleukin 17A	Proinflammatory cytokine involved in infectious diseases, inflammation, and autoimmune disorders	-1.43	1.11	0.75
*IL17F*	Interleukin 17F	Stimulates the production of several other cytokines	↓ -3.01	0.85	0.66
*IL17RA*	Interleukin 17 receptor A	Receptor for IL17A and IL17F	-0.31	-0.06	-0.13
*IL17RC*	Interleukin 17 receptor C	Receptor for IL17A and IL17F	-0.64	-0.04	0.37
*IL6R*	Interleukin 6 receptor	Forms a receptor complex with IL6R and IL6 signal transducer	-1.03	-0.58	-0.06
*TGFB1*	Transforming growth factor beta 1	TGFB1–3 encode a secreted ligand that activates and recruits SMAD family transcription factors	0.2	-0.15	-0.24
*TGFB2*	Transforming growth factor beta 2		0.31	0.6	0.71
*TGFB3*	Transforming growth factor beta 3		0.61	0.08	0.06
*TGFBR1*	Transforming growth factor beta receptor 1	TGFBR1–2 transduce the TGFB1, TGFB2, and TGFB3 signals from the cell surface to the cytoplasm	0.07	0.28	0.24
*TGFBR2*	Transforming growth factor beta receptor 2		0.54	0.1	0.04
*TLR4*	Toll-like receptor 4	Acts as a single transmembrane cell-surface receptor mediating innate immune responses	-0.5	-0.44	1
*IL23A*	Interleukin 23 subunit alpha	Functions as a heterodimeric cytokine with roles in innate and adaptive immunity and Th17-cell expansion	0.94	1.8	1.33
*IL23R*	Interleukin 23 receptor	Binds IL23 and mediates T-cell stimulation through activation of the Jak-Stat signaling cascade	1.49	1.35	-0.01
*IL12RB1*	Interleukin 12 receptor subunit beta 1	Part of the IL12R complex and associates with IL23R	-0.89	0.54	-0.16
*RORC*	RAR-related orphan receptor C	Belongs to the nuclear receptor family of transcription factors expressed by immune cells	0.14	0.09	-0.77
*STAT3*	Signal transducer and activator of transcription 3	Serves as a mediator of expression for various genes in response to cell stimuli such as viral or bacterial infections	0.45	0.16	0.06
*IRF4*	Interferon regulatory factor 4	Involved in interferon regulation in response to infection and interferon-inducible genes	0.6	-0.15	-0.35
*TRAF6*	TNF receptor-associated factor 6	Mediates signal transduction through activation of adapter protein complex 1 (AP-1) and NF-ĸB	0.43	0.15	0.02
*MAP3K7CL*	MAP3K7 C-terminal like	Represents a protein-coding gene located in the nucleus	1.23	-0.88	-1.14
*FOS*	Fos proto-oncogene, AP-1 transcription factor subunit	Acts as a regulator of cell proliferation, differentiation, and transformation	1.46	0.01	-0.32
*JUN*	Jun proto-oncogene, AP-1 transcription factor subunit	Forms a heterodimer with the FOS family to form the AP-1 transcription complex	0.7	0.19	0.22
*CEBPB*	CCAAT enhancer binding protein beta	Acts as a key regulator of genes involved in immune and inflammatory responses	0.57	0.54	0.65

FC: Fold change; (↑): Upregulated. (↓): Downregulated, |FC| ≥2, *p* <0.05.

### Absence of direct IL-17A process association found in target gene function analysis during *R*. *anatipestifer* stimulation on chicken splenic lymphocytes

**Fig 3:**
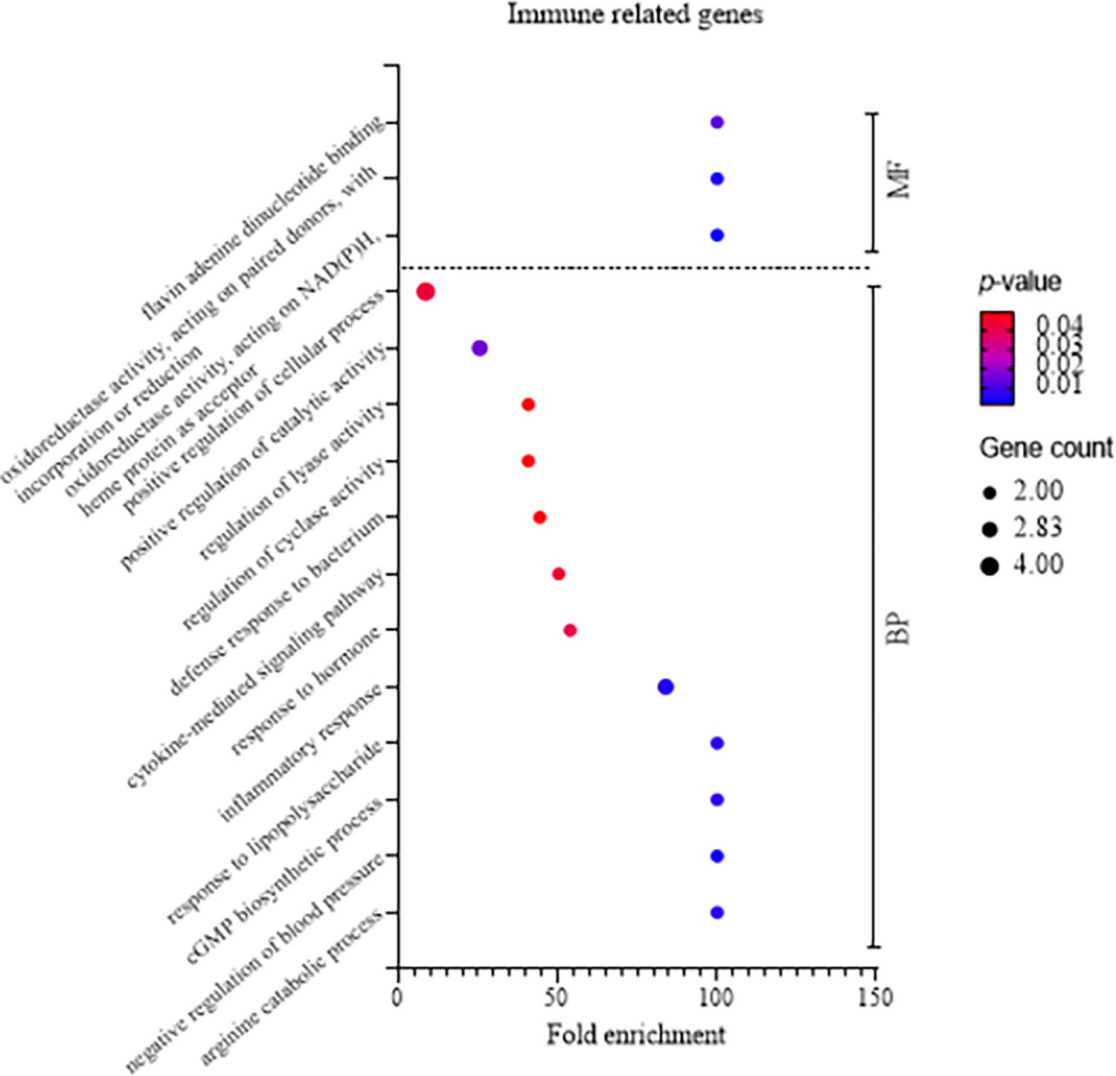
Summary of Gene Ontology (GO) enrichment analysis for NOS2 and CCL5 and their associated immune-related genes. GO enrichment analysis utilized an overrepresentation test to evaluate the significance of terms. A set of differentially expressed immune-related genes identified from RNA-seq, including NOS2, CCL5, and immune genes associated with their expression, was subjected to the analysis. Terms were categorized into Biological Process (BP) and Molecular Function (MF) using the *Gallus gallus* reference genome. The Fisher’s exact test and false discovery rate correction with a *p*-value threshold of <0.05 were applied. A bubble graph was generated to illustrate variations in fold enrichment, *p*-value, and gene count across the identified terms.

**Fig 4:**
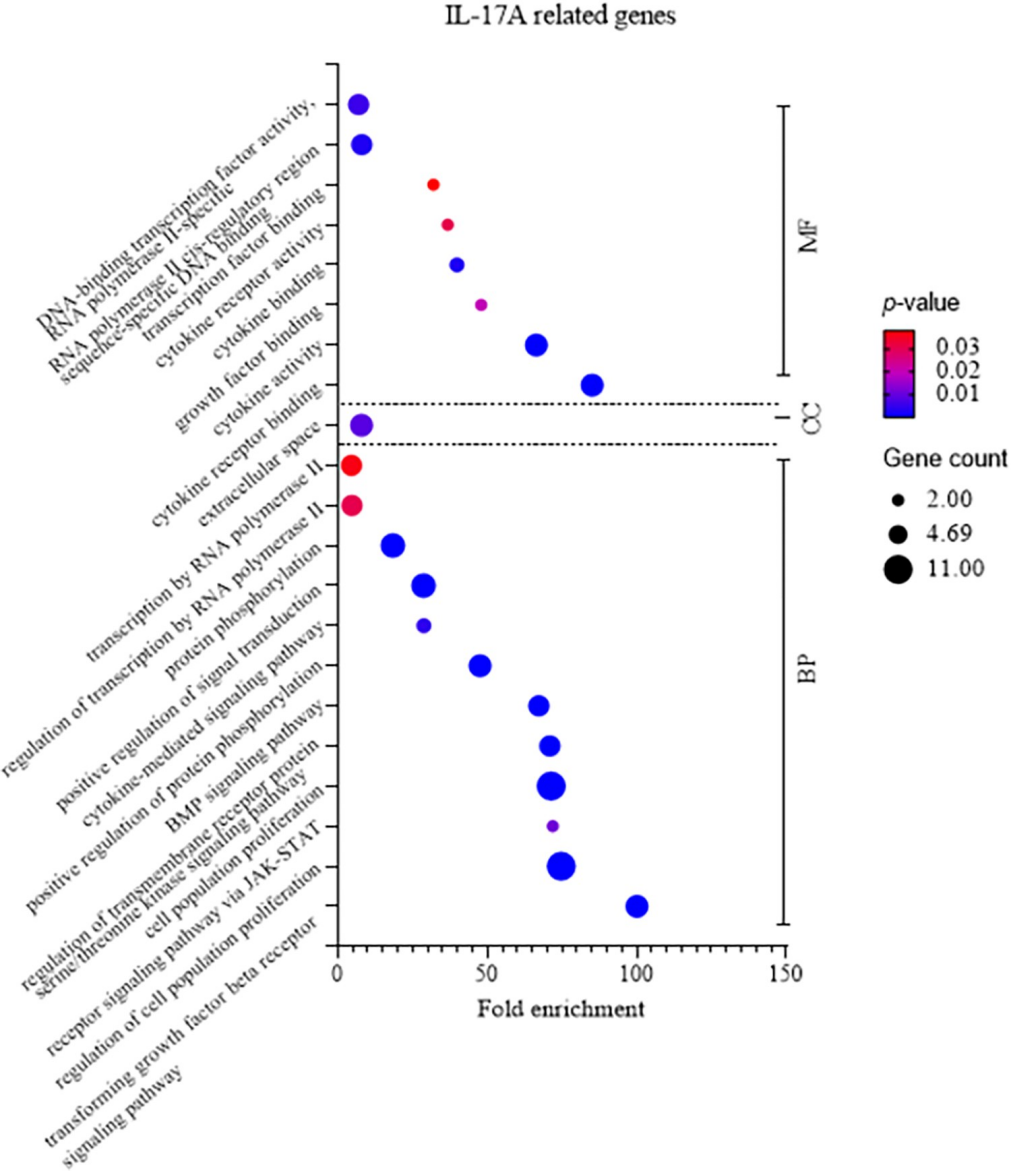
Summary of Gene Ontology (GO) enrichment analysis for IL-17A-related genes. GO enrichment analysis utilized an overrepresentation test to evaluate the significance of terms. The analysis utilized a set of differentially expressed IL-17A-related genes identified from RNA-seq. Terms were categorized into Biological Process (BP), Cellular Component (CC), and Molecular Function (MF) using the *Gallus gallus* reference genome. The Fisher’s exact test and false discovery rate correction with a *p*-value threshold of <0.05 were applied. A bubble graph was generated to illustrate variations in fold enrichment, *p*-value, and gene count across the identified terms.

GO enrichment analysis was conducted to explore the biological processes, cellular components, and molecular functions associated with immune-related terms. The analysis aimed to identify statistically overrepresented genes compared to the reference database list. The results revealed distinctive patterns for different gene sets. For NOS2, CCL5, and their associated genes, the predominant categories were positive regulation of cellular processes, positive regulation of catalytic activity, and inflammatory response (Fig 3). These terms primarily fall under the biological process category, emphasizing the active involvement of these genes in immune-related functions. NOS2, IL-6 and IL-1β in particular as expressed in Table 3, are either involved in inflammation or as pro-inflammatory cytokines. In contrast, the molecular function analysis of IL-17A-related genes demonstrated a higher gene count in terms of molecular function, cytokine receptor binding, and cytokine binding. The biological process categories with the highest gene count are regulation of cell population proliferation and cell population proliferation (Fig 4). Likewise, FOS2 in Table 4 functions as gene for cell population proliferation. This observation suggests that the GO analysis terms for IL-17A-related genes may not be directly aligned with the inflammatory processes of immune responses, unlike the GO terms associated with NOS2, CCL5, and their related genes.

### miR-456-3p expression is linked to NOS2 expression and miR-16-5p expression is linked to CCL5 expression

From the RNA-seq analysis, significant DEGs were identified, many of which are immune-related and exhibited upregulation upon stimulation of chicken splenic lymphocytes with *R*. *anatipestifer* at various time points. These DEGs include IL-6, IL-1β, IL-1R2, IL-4I1, CXCLi1, CXCLi2, IL-10, IL-13RA2, and IL-17C (Table 3). Using miRDB, gene target prediction was performed by matching the RNA-seq analysis list with the significant DE-miRNAs. This established an association between specific miRNAs and target genes. For example, miR-456-3p was linked to NOS2, and miR-16-5p was linked to CCL5 (Table 5). Further investigation revealed that the downregulated expression of miR-456-3p, with a log_2_ fold change at 8 and 24 hours corresponds to upregulated NOS2 expression with a positive log_2_ fold change at the same time points. Conversely, downregulated expression of miR-16-5p, with a log_2_ fold change at 4 hours, corresponds to upregulated CCL5 expression at the same time point. This observation aligns with the concept that miRNAs lead to mRNA degradation, implying that downregulated miRNAs result in increased mRNA expression. To further investigate the interaction sites between NOS2 and CCL5 regions and their respective binding miRNAs (miR-456-3p and miR-16-5p), RNA hybrid analysis was used (S1 Fig). The outcomes of this prediction underscore the molecular mechanisms involving miRNA-mediated gene regulation.

**Table 5:** Summary of NOS2 and CCL5 expression and their corresponding miRNAs.

Significantly DE-mRNA		Log_2_ FC (4 h)	Log_2_ FC (8 h)	Log_2_ FC (24 h)	Matched significant DE-miRNA	Log_2_ FC (4 h)	Log_2_ FC (8 h)	Log_2_ FC (24 h)
**NOS2**	↑ 3.41		↑ 3.18	↑ 3.09	**gga-miR-456-3p**	↑ 2.26	-1.6	-1.66
**CCL5**	↑ 2.02		1.74	0.38	**gga-miR-16-5p**	-1.8	↑ 2.19	1.23

DE: Differentially expressed; FC: Fold change.

(↑): Upregulated. (↓): Downregulated, |FC| ≥2, *p* <0.05.

### qRT-PCR analysis confirms RNA-seq results for IL-17A and IL-17F expression regulation

**Fig 5:**
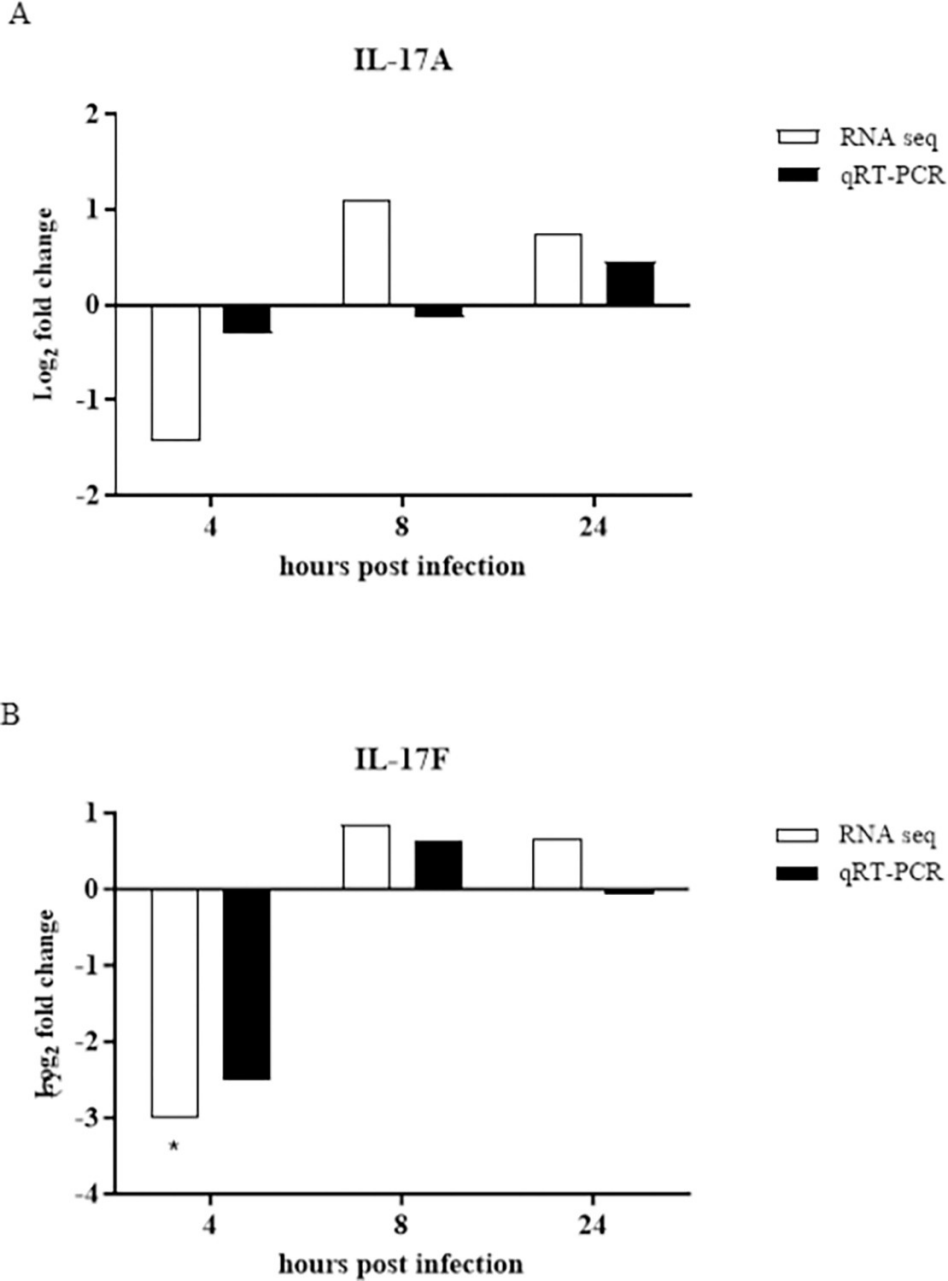
Validation of RNA-seq results through qRT-PCR analysis. Chicken splenic lymphocytes were cultured to confluency at 5 × 10^6^ cells per well, stimulated with 1 × 10^6^ heat-killed *R*. *anatipestifer* CFUs per well, and collected at 4, 8, and 24 hours. The experiment was conducted in triplicate, and pooled samples were subjected to RNA-seq analysis. qRT-PCR validation of the same samples was performed in triplicate. The expression of (A) IL-17A and (B) IL-17F was compared and validated by qRT-PCR. Validation was done from comparing KRA stimulated cells vs. negative control at each corresponding time point and for both RNA-seq and qRT-PCR result. * |FC| ≥2, *p* <0.05.

Two mRNAs, IL-17A and IL-17F, were subjected to qRT-PCR analysis (Fig 5). Expression of both IL-17A and IL-17F was downregulated at 4 hours post-infection in both RNA-seq and qRT-PCR analyses, displaying minimal variation. At the 8-hour time point, qRT-PCR revealed reduced expression of IL-17A, whereas RNA-seq showed enhanced expression. Conversely, RNA-seq and qRT-PCR indicated upregulation of IL-17F expression. At 24 hours post-infection, IL-17A expression was upregulated across both methods, while IL-17F expression was upregulated in RNA-seq and downregulated in qRT-PCR, displaying minimal variation. Overall, IL-17A and IL-17F expression, unless downregulated, maintained very low to moderate levels. These results validate the RNA-seq results.

### qRT-PCR validation of the interactions between miR-456-3p and NOS2 and miR-16-5p and CCL5

**Fig 6:**
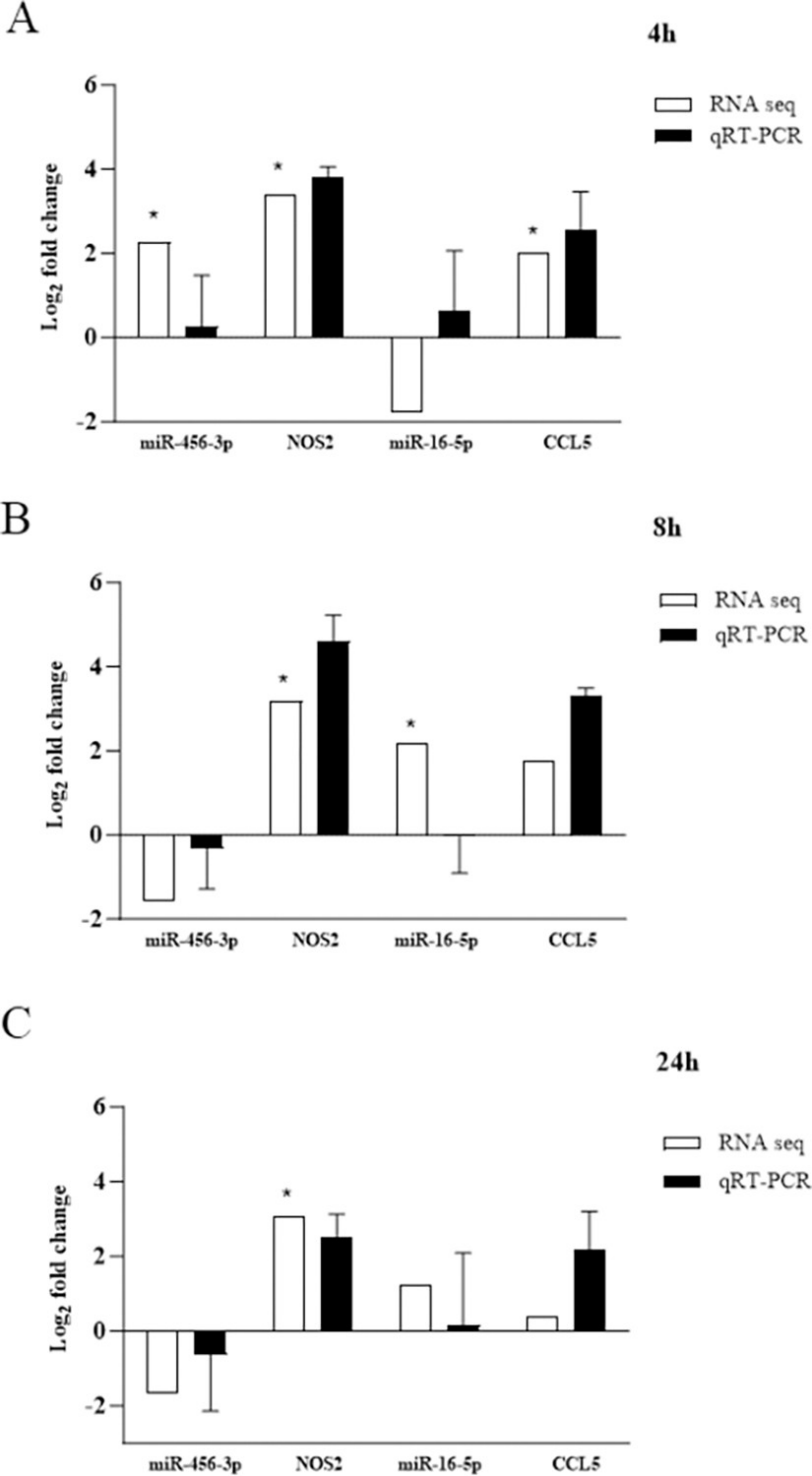
Validation of miRNA/mRNA interactions using qRT-PCR. Chicken splenic lymphocytes were cultured to confluency at 5 × 10^6^ cells per well, stimulated with 1 × 10^6^ heat-killed *R*. *anatipestifer* CFUs per well, and collected at 4, 8, and 24 hours. The experiment was conducted in triplicate, and pooled samples were subjected to miRNA-seq and RNA-seq analyses. qRT-PCR of the same samples was performed in triplicate. The expression of miR-456-3p in relation to NOS2 and miR-16-5p in relation to CCL5 was validated by qRT-PCR at (A) 4, (B) 8, and (C) 24 hours. Validation was done from comparing KRA stimulated cells vs. negative control at each corresponding time point and for both RNA-seq and qRT-PCR result. * |FC| ≥2, *p* <0.05.

Two miRNAs, miR-456-3p and miR-16-5p, along with their respective target mRNAs, NOS2 and CCL5, underwent qRT-PCR analysis. An inverse correlation between miR-456-3p and NOS2 was evident at 8 hours and 24 hours, with miR-456-3p being downregulated and NOS2 being upregulated (Fig 6). Interestingly, miR-16-5p, predicted to be downregulated and inversely correlated with CCL5, exhibited a positive correlation in the qRT-PCR analysis. These findings provided corroborative evidence for the accuracy of both RNA-seq and smRNA-seq methodologies.

### Th17 cells are poorly or negatively expressed in chicken splenic lymphocytes stimulated with *R*. *anatipestifer*

**Fig 7:**
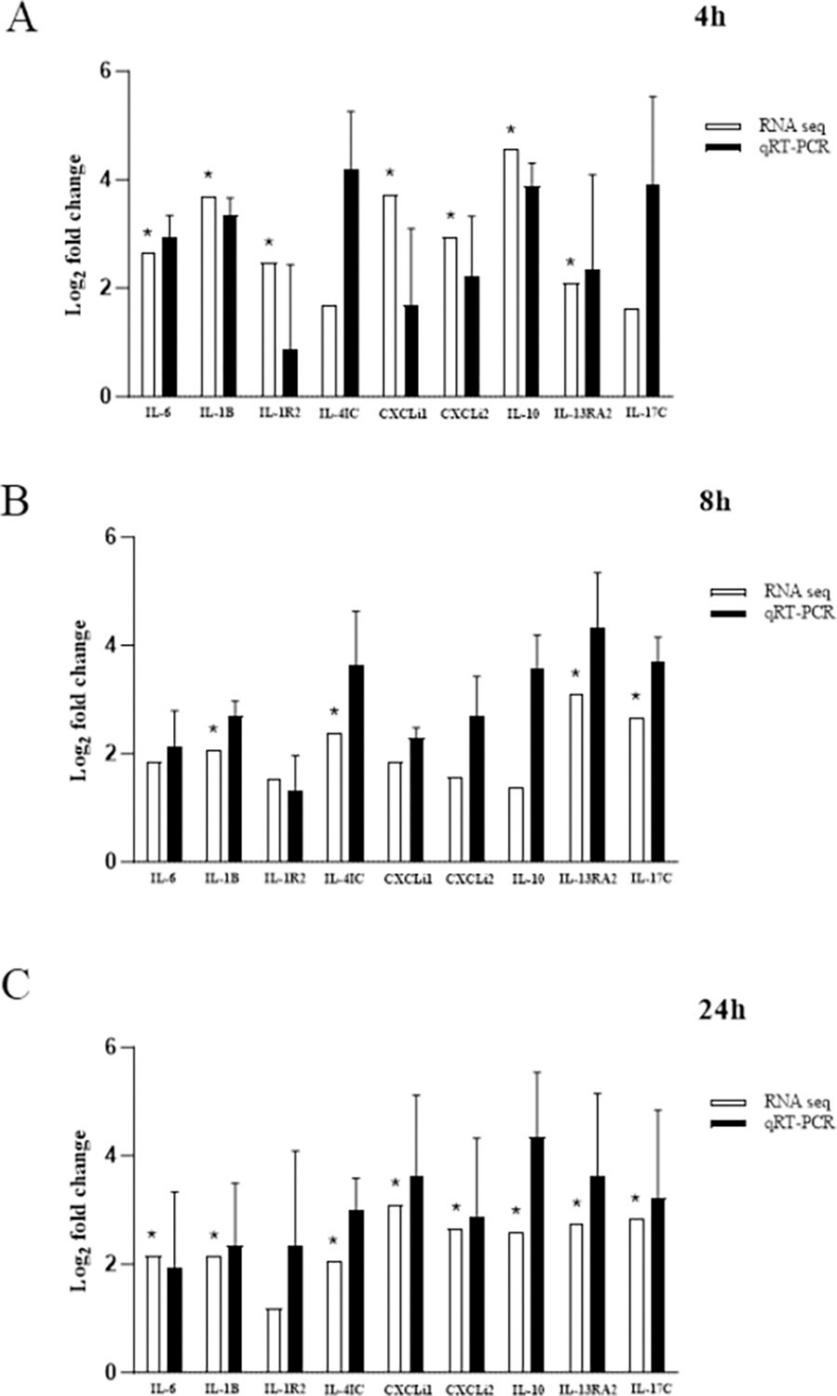
Validation of immune gene expression associated with NOS2 and CCL5 mRNA targets using qRT-PCR. Chicken splenic lymphocytes were cultured to confluency at 5 × 10^6^ cells per well, stimulated with 1 × 10^6^ heat-killed *R*. *anatipestifer* CFUs per well, and collected at 4, 8, and 24 hours. The experiment was performed in triplicate, and pooled samples were subjected to RNA-seq analysis. qRT-PCR of the same samples was performed in triplicate. Expression of immune-related genes associated with NOS2 and CCL5, including IL-6, IL-1β, IL-1R2, IL-4I1, CXCLi1, CXCLi2, IL-10, IL-13RA2, and IL-17C, at (A) 4, (B) 8, and (C) 24 hours was validated by qRT-PCR. Validation was done from comparing KRA stimulated cells vs. negative control at each corresponding time point and for both RNA-seq and qRT-PCR result. * |FC| ≥2, *p* <0.05.

Common immune-related cytokines associated with NOS2 and CCL5 were subjected to qRT-PCR validation. Similar expression trends were observed for IL-6, IL-1β, IL-1R2, IL-4IC, CXCLi1, CXCLi2, IL-13RA2, and IL-17C at 4-, 8-, and 24-hours post-R. *anatipestifer* stimulation (Fig 7). These qRT-PCR results corroborate the reliability of the RNA-seq findings. These findings indicated that for both RNA-seq and qRT-PCR results showed a moderate to low log_2_ fold change expression of each respective cytokine. Also, Th17 cells are either not too highly expressed if not downregulated, leading to a diminished Th17 response (Table 3).

### miR-456-3p and miR-16-5p mimics cause downregulation of related genes

**Fig 8:**
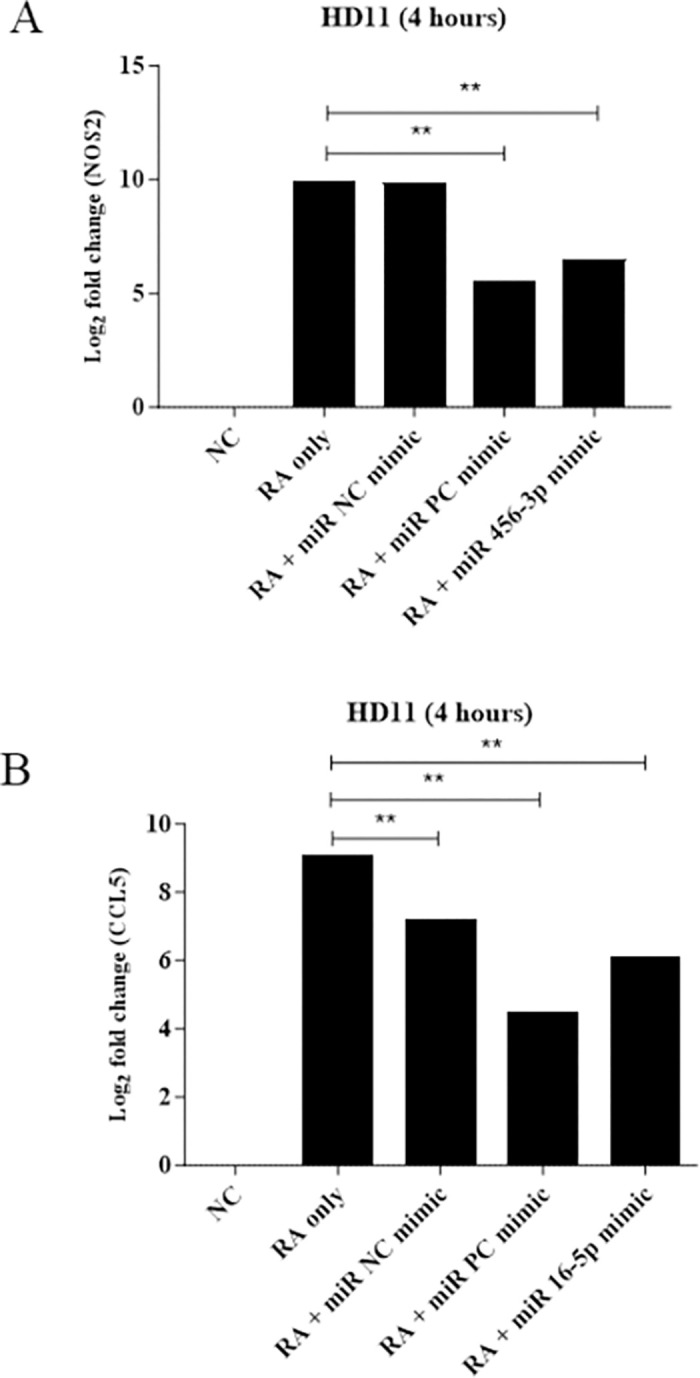
Functional validation of the miRNA mimics by qRT-PCR. HD11 cells were seeded overnight in 6-well plates at a density of 5 × 10^6^ cells/well, achieving a minimum confluency of 80%. Cells were then stimulated with 1 × 10^6^ heat-killed *R*. *anatipestifer* CFUs per well and simultaneously transfected with either the miR-456-3p or miR-16-5p mimics. Samples were collected and analyzed after 4 hours. For comparison, the miR-NC mimic and the miR-PC mimic were employed. The HD11 cells were transfected with either (A) the miR-456-3p mimic or (B) the miR-16-5p mimic and then collected and analyzed after 4 hours. (RA) *R*. *anatipestifer*, (NC) negative control, (PC) positive control. ** *p* <0.01.

In HD11 cells, *R*. *anatipestifer* stimulation alone led to the upregulation of NOS2 and CCL5. Similarly, treatment with *R*. *anatipestifer* plus a negative control miRNA mimic exhibited comparable values. Transfection of the miR-456-3p mimic into *R*. *anatipestifer–*stimulated HD11 cells resulted in the simultaneous downregulation of NOS2 and CCL5 (Fig 8). Interestingly, treatment with *R*. *anatipestifer* plus a positive control miRNA mimic exhibited comparable values as well. These findings suggest that transfection of *R*. *anatipestifer–*stimulated HD11 cells with the miR-456-3p and miR-16-5p mimics effectively inhibited the typical upregulation of NOS2 and CCL5, respectively, observed during *R*. *anatipestifer* stimulation alone.

## Discussion

In this study, we employed next-generation sequencing and data analysis to annotate the response of chicken splenic lymphocytes to *R*. *anatipestifer* stimulation, both in the presence and absence of the pathogen. Our focus was directed toward IL-17A, which was not significantly expressed. Subsequent examination of IL-17A-related genes revealed a lack of significant expression, except for IL-17F, which was downregulated at the 4-hour time point post-infection. Throughout all time points, we observed consistent downregulation of IL-6R, a precursor associated with Th17 cell proliferation, as well as the receptor for IL-17A and IL-17F, IL-17RA. These findings raise the possibility that the subdued expression of these key receptors may contribute to the limited Th17 response during *R*. *anatipestifer* infection in chickens. Additionally, both IL-17A and IL-17F were downregulated at the 4-hour time point, further substantiating the finding that the majority of IL-17A-related genes exhibited moderate to reduced expression across the different time points. This observed pattern may contribute to the diminished Th17-driven response during *R*. *anatipestifer* infection in chickens.

Our findings align with a study conducted by Fernandez et. al. [5], which indicates that the expression of Th2 phenotypes results in the suppression of IL-17A expression in chicken. Notably, chicken splenic lymphocytes exhibited heightened expression of Th1 and Th2 phenotypes following *R*. *anatipestifer* stimulation. T helper cells are classified into various subsets, including Th1, Th2, Th17, and T regs, based on their production of immunoregulatory cytokines. While Th1 is associated with cellular immune responses and Th2 with humoral immunity [41], our observed differential gene expression profile, particularly the subdued or absent expression of the Th17 molecule IL-17A, is in agreement with Fernandez et al.’s findings [5]. Furthermore, the elevated expression of IL-1β and IL-6 in conjunction with the lack of IL-17A upregulation suggests that the predominant immune response triggered by *R*. *anatipestifer* infection in chickens does not primarily stem from a Th17-driven mechanism.

I*n silico* transcriptional analysis was performed to identify DE-mRNAs and DE-miRNAs during *R*. *anatipestifer* stimulation. By combining and comparing the data, miRDB was used to match target miRNAs from the significant DE-miRNAs. The results established a correlation between NOS2 and miR-456-3p, as well as CCL5 and miR-16-5p. Further examination involved GO enrichment analysis of the targeted mRNAs, revealing their predominant involvement in inflammatory responses and other biological processes. These findings align with a study conducted by Liu et. al. (2015), which demonstrated that *R*. *anatipestifer* infection promotes NOS2 activity [8]. Consequently, the upregulation of NOS2 expression triggers the upregulation of other genes, particularly those associated with Th1 and Th2 responses, while showing limited impact on Th17-related genes in the context of *R*. *anatipestifer* infection in chickens.

In the context of the GO enrichment analysis, although the molecular function category is related to cytokine binding, the biological process category predominantly highlights cell population proliferation. Interestingly, the other biological processes do not exhibit a direct correlation with the inflammatory processes or immune responses, which are fundamental aspects of GO enrichment terms. This observation implies that IL-17A-related genes might not actively contribute to the inflammatory or immune responses triggered by *R*. *anatipestifer* stimulation in chicken splenic lymphocytes. The utilization of GO enrichment analysis provides valuable insights into the consequences of altered gene expression levels, offering a deeper understanding of cellular components, molecular functions, and biological processes associated with the specific set of genes under investigation [42].

Inflammation is a pivotal response within the host defense system against infections and injuries. A seminal study by Harris et. al. [43] established a connection between miRNAs and inflammation. Their study highlighted the role of miR-126 in mediating leukocyte adherence to endothelial cells through the inhibition of vascular cell adhesion molecule-1 (VCAM-1) expression, thus regulating vascular inflammation. Another significant model linking miRNAs with inflammation resulted from a study by Sonkoly et. al. [44], which centered on skin inflammation in psoriatic skin models. This study demonstrated the consequences of elevated miR-203 levels, which led to reduced cytokine signaling 3 (SOCS3) expression. As a result, sustained activation of the signal transducer and activator of transcription 3 (STAT3) signaling pathway occurred, subsequently contributing to the prolonged state of skin inflammation.

This study confirms the association between miR-456-3p and NOS2 through qRT-PCR validation, affirming the findings from both smRNA-seq and RNA-seq analyses. Prior research has explored functional aspects of chicken miRNAs, and reports indicate potential regulatory roles for both miR-456-3p and miR-16-3p in immune-related genes. Specifically, miR-456-3p shows increased expression during dexamethasone-induced immunosuppression [16], a slight increase during infectious bronchitis virus infection in primary chicken dendritic cells [17], and reduced expression in avian influenza-infected chicken lungs [18]. Meanwhile, studies involving miR-16-5p have indicated the effects of its elevated expression on the PI3K/Akt/NF-ĸB pathway, leading to an anti-inflammatory effect [19], and its downregulation contributing to CD4+ T-cell activation [20]. In relation to the immune response during *R*. *anatipestifer* infection in ducks, a previous study [45] demonstrated the expression of IL-17A in both CD4+ and CD4- cells.

Through transfection experiments, this study further validated the associations between miR-456-3p and NOS2, as well as miR-16-5p and CCL5. In response to *R*. *anatipestifer* stimulation, HD11 cells exhibited upregulated expression of NOS2 and CCL5. A similar pattern was observed when utilizing a negative control miRNA mimic, which had sequences distinct from miR-456-3p and miR-16-5p. Conversely, transfection with the miR-456-3p mimic resulted in downregulation of NOS2. Similarly, transfection with a miR-16-5p mimic led to downregulation of CCL5. The positive control mimic, with similar sequences to the miRNA mimics, displayed reduced expression of NOS2 and CCL5. These findings align with the concept that miRNA downregulation corresponds to gene expression upregulation.

Additionally, the inflammatory response may act as a factor in the pathogenic impact of riemerellosis. Therefore, these results suggest that promotion of Th1 or Th2 cytokines or production of NOS2 or CCL5 has a protective effect against *R*. *anatipestifer*. Thus, miRNAs miR-456-3p and miR-16-5p can be used for therapeutic intervention.

In conclusion, this study identified miR-456-3p and miR-16-5p as potential regulators of the inflammatory process during *R*. *anatipestifer* stimulation in chicken splenocytes. It also established NOS2 and CCL5 as their respective target genes, thereby elucidating the associated inflammatory cytokines influenced by these miRNAs. Furthermore, we confirmed the increased expression of Th1- and Th2-associated cells, while observing only a moderate Th17-cell response during *R*. *anatipestifer* stimulation in chicken splenocytes. This investigation sheds light on the intricate immune mechanisms at play during the disease course, highlighting the absence of a robust Th17 immune response and the roles played by miRNAs in this context. While this study offers valuable insights into the immune response in chickens, future research could benefit from a comparative analysis involving ducks, which are the primary hosts for *R*. *anatipestifer*. Also, ducks would be a better candidate to further understand *R*. *anatipestifer*, as the disease itself have been established to upregulate IL-17A both invivo and invitro. Such comparisons could enhance our understanding of the specific role of IL-17A in the context of riemerellosis and provide a more comprehensive view of the underlying mechanisms. Additionally, miRNA studies in ducks might also help in identifying gene regulation to control riemerellosis.

## Supporting information

S1 Fig Predicted interactions between target mRNAs and their corresponding miRNAs. Utilizing miRDB, gene target prediction was performed, and the resulting miRNA and mRNA sequences were input into the RNA hybrid tool for analysis. This approach facilitated the prediction of targeting sites through base pairing. The RNA hybrid analysis generated predicted base pair interactions involving (A) NOS2 and miR-456-3p, and (B) CCL5 and miR-16-5p. Colors indicated red for mRNA, green for miRNA.
(TIF)

S1 Table List of differentially expressed common immune-related gene from RNA-seq results. (DOCX)

S2 Table Summary of differentially expressed genes from RNA-seq results. (XLSX)
