# Supplementary figures and images for: Promotion of Th1 and Th2 responses over Th17 in Riemerella anatipestifer stimulation in chicken splenocytes: Correlation of gga-miR-456-3p and gga-miR-16-5p with NOS2 and CCL5 expression
Source: PLoS One. 2023 Nov 6;18(11):e0294031. doi: 10.1371/journal.pone.0294031 (PMC10627459; doi:10.1371/journal.pone.0294031)

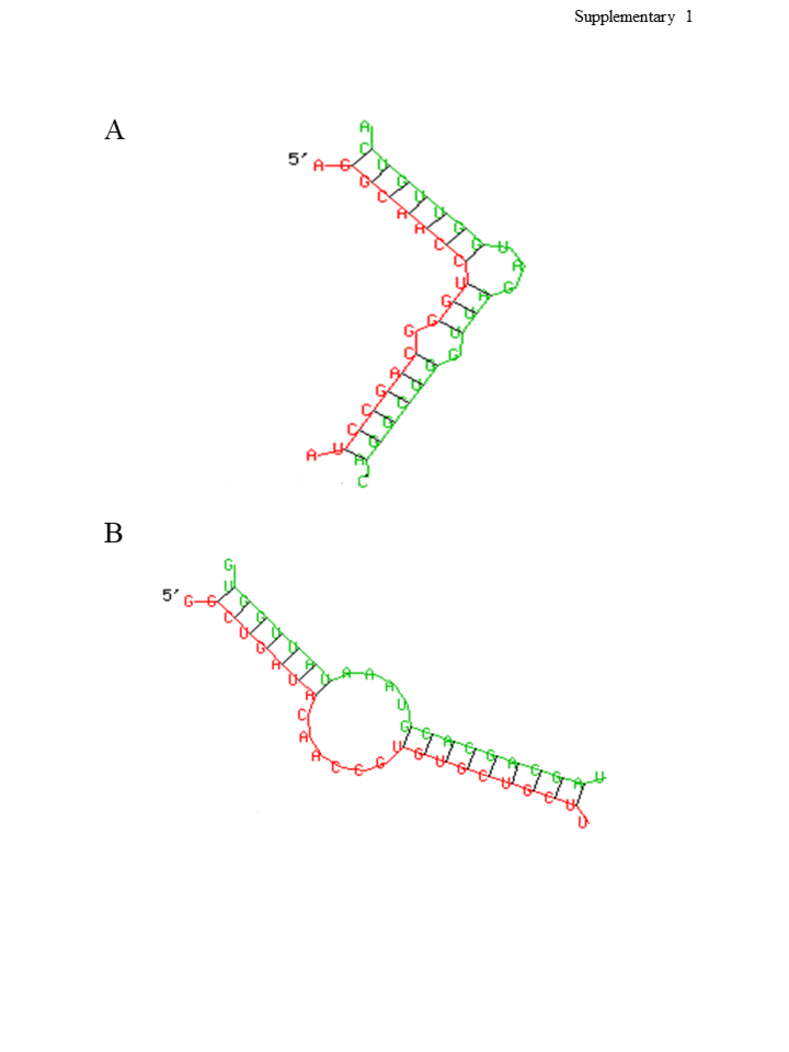

Supplement: S1 Fig — Utilizing miRDB, gene target prediction was performed, and the resulting miRNA and mRNA sequences were input into the RNA hybrid tool for analysis. This approach facilitated the prediction of targeting sites through base pairing. The RNA hybrid analysis generated predicted base pair interactions involving (A) NOS2 and miR-456-3p, and (B) CCL5 and miR-16-5p. Colors indicated red for mRNA, green for miRNA. (TIF) [file pone.0294031.s001.tif]
