# Supplementary material for: Promotion of Th1 and Th2 responses over Th17 in Riemerella anatipestifer stimulation in chicken splenocytes: Correlation of gga-miR-456-3p and gga-miR-16-5p with NOS2 and CCL5 expression
Source: PLoS One. 2023 Nov 6;18(11):e0294031. doi: 10.1371/journal.pone.0294031 (PMC10627459; doi:10.1371/journal.pone.0294031)
Supplement: S1 Table — (DOCX) [file pone.0294031.s002.docx]

**Supplementary Table 1. List of differentially expressed common immune-related genes from RNA-seq results.**

| **Gene** | **Description** | **Function** | **Log2FC(4h)** | **Log2FC(8h)** | **Log2FC(24h)** |
| --- | --- | --- | --- | --- | --- |
| *LOC422654* | Chemokine (C-X-C motif) ligand 1-like | Chemoattractant of neutrophils or other non-hematopoietic cells to the site of infection | ↑3.97 | ↑ 3.87 | ↑ 4.27 |
| *NOS2* | Nitric oxide synthase-2 | Involved in inflammation by enhancing synthesis of proinflammatory mediators like IL6 and IL8 | ↑ 3.41 | ↑ 3.18 | ↑ 3.09 |
| *TNIP3* | TNFAIP3-Interacting Protein 3 | Inhibits NF-kappa-B activation induced by TNF, TLR4 and IL-1. Also involved in IL-23 signaling pathway | ↑ 2.42 | ↑ 2.41 | ↑ 2.85 |
| *BATF3* | Basic Leucine Zipper ATF-Like Transcription Factor 3 | Regulates CD8+ differentationa and produce IL-12 during infection | ↑ 2.05 | ↑ 2.21 | 0.85 |
| *FLT1* | Fms Related Receptor Tyrosine Kinase 1 | Mediates MAP kinase signaling pathway, plays essential role for macrophage function and chemotaxis | ↑ 2.26 | ↑ 2.07 | 1.74 |
| *LOC395551* | Chemokine (C-C motif) ligand 4 | Involved in chemotactic activity, involved in cellular response to IL-1, IFNy and TNF | ↑ 2.4 | 1.02 | 1.54 |
| *CCLL4* | C-C motif chemokine | Involved in chemotactic activity, involved in cellular response to IL-1, IFNy and TNF | ↑ 5.63 | 1.97 | ↑ 3.28 |
| *LOC419276* | Bactericidal permeability-increasing protein | Antibacterial response | ↑ 4.03 | ↑ 3.19 | ↑ 3.51 |
| *UMODL1* | Uromodulin Like 1 | Facilitates neutrophil migration across renal epithelia, receptor for binding and endocytosis of IL-1, IL-2 and TNF | 1.14 | ↑ 2.41 | ↑ 2.45 |
| *CXCL14* | C-X-C Motif Chemokine Ligand 14 | Homeostasis of monocyte-derived macrophages | ↑ 2.24 | ↑ 2.68 | ↑ 2.3 |
| *LOC770026* | OX-2 membrane glycoprotein-like | Negative regulation of leukocyte, negative regulation of macrophage activation | ↑ 2.03 | 1.02 | 0.91 |
| *CCL5* | C-C Motif Chemokine Ligand 5 | Chemoattractant of monocytes, memory T cells and eosinophils | ↑ 2.02 | 1.74 | 0.38 |
| *DUSP8* | Dual Specificity Phosphatase 8 | Negatively regulates MAPK pathway | ↑ 2.31 | 1.24 | ↑ 2.09 |
| *VNN2* | Vanin 2 | Involved in the thymus homing of bone marrow cells, migration and motility of neutrophil | ↓ -3.03 | -1.35 | -1.47 |
| *PTGS2* | Prostaglandin-Endoperoxide Synthase 2 | Cyclooxygenase, cytokine signaling | ↑ 2.35 | 1.6 | 0.9 |
| *CD72L2* | CD72 molecule like 2 | B-cell differentiation antigen CD72-like | 0.61 | 1.16 | ↑ 2.36 |
